# Management of two circulations in a COVID‐19 patient with secondary superinfection

**DOI:** 10.14814/phy2.15602

**Published:** 2023-02-17

**Authors:** Rachael Stadlen, Arun K. Singhal, Robert M. Reed, Jeffrey D. Hasday, Melissa L. Bates, Gregory A. Schmidt, Michael Eberlein

**Affiliations:** ^1^ Division of Pulmonary, Critical Care and Occupational Medicine University of Iowa Iowa City Iowa USA; ^2^ Department of Thoracic and Cardiovascular Surgery University of Iowa Iowa City Iowa USA; ^3^ Division of Pulmonary and Critical Care Medicine University of Maryland Baltimore Maryland USA; ^4^ Department of Health and Human Physiology University of Iowa Iowa City Iowa USA; ^5^ Department of Pediatrics University of Iowa Iowa City Iowa USA; ^6^ Department of Internal Medicine University of Iowa Iowa City Iowa USA

**Keywords:** ARDS, cooling, COVID‐19, shunt equation, VV‐ECMO

## Abstract

Optimal oxygenation in the intensive care unit requires adequate pulmonary gas exchange, oxygen‐carrying capacity in the form of hemoglobin, sufficient delivery of oxygenated hemoglobin to the tissue, and an appropriate tissue oxygen demand. In this Case Study in Physiology, we describe a patient with COVID‐19 whose pulmonary gas exchange and oxygen delivery were severely compromised by COVID‐19 pneumonia requiring extracorporeal membrane oxygenation (ECMO) support. His clinical course was complicated by a secondary superinfection with staphylococcus aureus and sepsis. This case study is provided with two goals in mind (1) We outline how basic physiology was used to address life‐threatening consequences of a novel infection—COVID‐19. (2) We describe a strategy of whole‐body cooling to lower the cardiac output and oxygen consumption, use of the shunt equation to optimize flow to the ECMO circuit, and transfusion to improve oxygen‐carrying capacity when ECMO alone failed to provide sufficient oxygenation.

## Graphical abstract

COVID‐19 pneumonia can require extracorporeal membrane oxygenation (ECMO) support. The use of the shunt analogy and the shunt equation can be an important tool to analyze and optimize relative blood flow to the ECMO circuit and oxygenation. We describe a strategy of whole‐body cooling to lower the cardiac output and oxygen consumption in a COVID‐19 patient with critical hypoxia on VV‐ECMO.

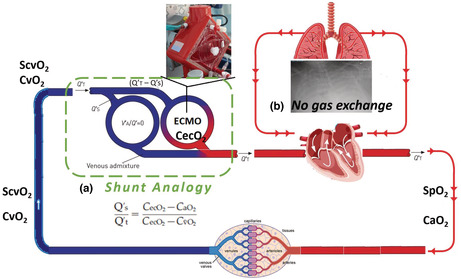

## 1 CLINICAL CASE

A 41‐year‐old male patient with no significant medical history was admitted to the Intensive Care Unit with progressive respiratory failure one week after diagnosis with COVID‐19. He was intubated, started on neuromuscular blockade, and ventilated in the prone position.

Despite intubation and prone ventilation with 100% oxygen, the patient remained hypoxemic (po_2_ = 65 mmHg) and hypercarbic (pco_2_ = 60 mmHg). He was cannulated for veno‐venous extracorporeal membrane oxygenation (VV‐ECMO) with bi‐femoral access. A 21‐French multistage drainage cannula was inserted with the tip in the infra‐hepatic inferior vena cava. A 25‐French multistage return cannula was inserted with tip at the level of the right atrium. The initial ECMO blood flow was 3 liters per minute (L/min) and a sweep gas flow of 3 L/min, resulting in a SaO_2_ of 92% and central venous oxygen saturation (SvO_2_) of 61%.

**FIGURE 1:**
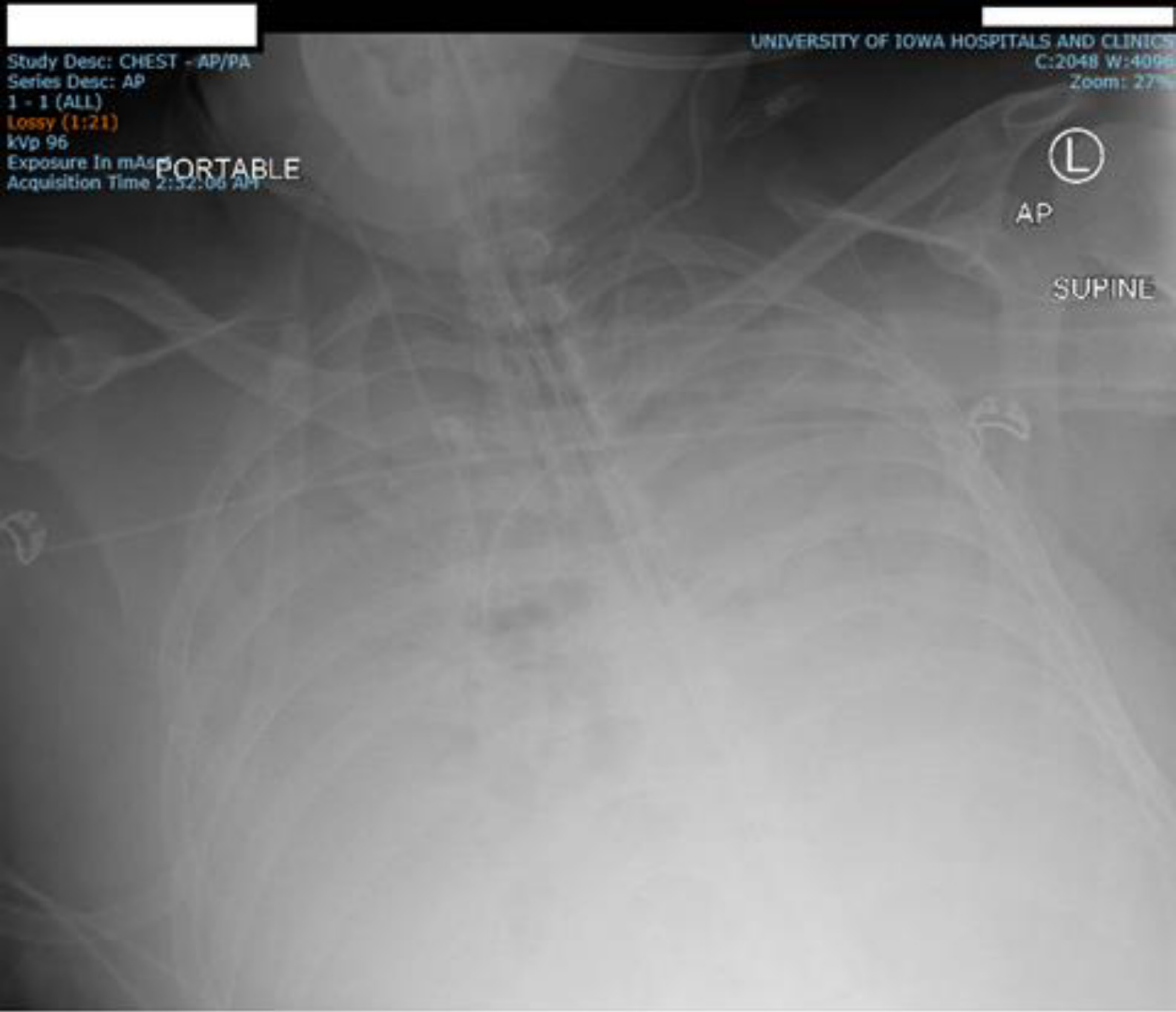
Chest X‐ray showing complete opacification of bilateral lung fields.

He developed distributive shock, with sinus tachycardia (up to 150 bpm) and hypotension requiring a norepinephrine infusion. A chest X‐ray revealed complete opacification of both lung fields (Figure 1). Respiratory and blood cultures grew Methicillin‐sensitive Staphylococcus aureus (MSSA). A transthoracic echocardiogram (TTE) demonstrated an ejection fraction of 73% with normal left ventricular systolic function and no valvular pathology. Tricuspid annular plane systolic excursion was 27 mm, indicating normal right ventricular function. An arterial blood gas revealed pH 7.51, PaCO_2_ 45 mmHg, and PaO_2_ 39 mmHg. His ECMO settings were maximized with a circuit blood flow of 6 L/min and sweep gas flow of 15 L/min. There was no significant ECMO‐circuit recirculation, and central venous saturation (SvO_2_) was 45%.

**FIGURE 2:**
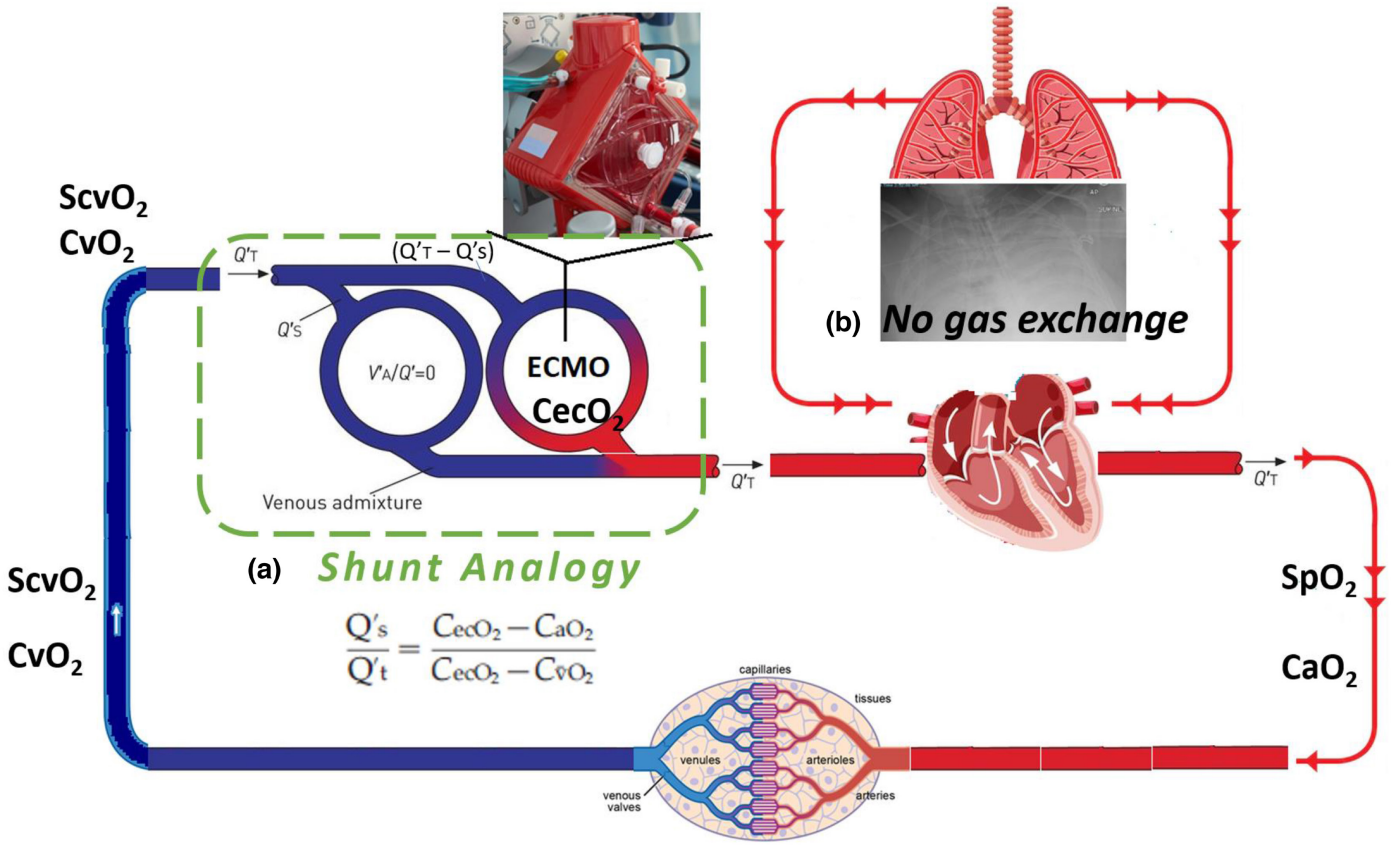
Illustration of the shunt analogy (a) occurring during VV‐ECMO support in setting of complete pulmonary failure (b) and absence of ECMO recirculation. All gas exchange occurs via the VV‐ECMO circuit. The native cardiac output [*Q*_T_] divides into two paths: (1) blood not drawn into the ECMO circuit and passing through as shunt flow [*Q*_s_] without oxygenation and ECMO‐circuit blood flow [*Q*_T_ − *Q*_s_] with oxygenation (2).

Despite maximal support, SaO_2_ was consistently between 73% and 82%, regardless of ventilator FIO_2_ of 0.3 or 1.0, indicating that all gas exchange was occurring within the ECMO circuit and there was negligible pulmonary gas exchange (Table 1 and Figure 2). Proning became difficult because it interrupted his ECMO flow. He remained sedated and paralyzed on pressure control ventilation with pressure control of +10 cm H_2_O above a PEEP of 10 cm H_2_O with a resulting tidal volume of 30–40 mL (respiratory system compliance of 3–4 mL/cm H_2_O). The patient had a lactate of 2.3 mmoL/L (normal 0.5–2.2 mmoL/L) and hemoglobin of 7.5 g/dL in the absence of active bleeding. Our efforts at improving pulmonary gas exchange with recruitment maneuvers, bronchoscopies to clear airways, and attempts to ventilate in the prone position were not successful. Complete pulmonary failure persisted.

**TABLE 1:** Patient, ventilator, and VV‐ECMO data at different time points, as indicated.

Time	ECMO Day 11—Baseline (time)			ECMO Day 11—cooling (time)	
	8:00–9:00	12:00–13:00	15:00–16:00	17:00–18:00	20:00–21:00
Patient					
Temp (°C)	36.7	36.8	36.8	35.9	35.9
HR (bpm)	98	96	104	94	87
BP (mmHg)	128/83	124/59	136/65	147/71	147/70
SaO_2_ (%)	75%	77%	74%	88%	89%
SvO_2_ (%)	48%	49%	45%	58%	63%
PaCO_2_(mmHg)			45		44
PaO_2_ (mmHg)			39		51
Hgb (g/dL)	7.5	7.5	7.5	7.5	7.5
CaO_2_			7.554		9.097
CvO_2_			4.62		6.43
Ventilator					
FiO_2_ (%)	***100%***	***30%***	30%	30%	30%
TV (ml)	35	34	35	32	
RR	15	15	15	15	15
et CO_2_ (mmHg)	0	0	0	0	0
VV‐ECMO					
Sweep (L)	15	15	15	15	15
Flow (L)			6	6	6
PecO_2_ (mmHg)			224		228
CecO_2_			10.72		10.74
Δ P (mmHg)			35	41	

Abbreviations: BP, blood pressure; CaO_2_, Oxygen content of arterial blood—calculation based on Equation (6) in main text; CecO_2_, “endcapillary” content of oxygen = post‐ECMO‐oxygenator content of oxygen; CvO_2_, oxygen content of venous blood; et, endtidal; FiO_2_, fraction of oxygen in inspired air; Hgb, hemoglobin; HR, heart rate in beats per minute (bpm); PaCO_2_, partial pressure of CO_2_ in arterial blood; PaO_2_, partial pressure of O_2_ in arterial blood; PecO_2_, “endcapillary” partial pressure of oxygen = post‐ECMO‐oxygenator partial pressure of oxygen; SaO_2_, saturation of arterial blood with oxygen; SvO_2_, saturation of central venous blood with oxygen; Temp, temperature in °C; TV, Tidal Volume; Δ P, delta pressure over ECMO membrane oxygenator.

Considering our patient's worsening, critical hypoxemia (SpO_2_ 73%–82%), we proceeded to cool the patient by 1°C to reduce oxygen consumption, increase SvO_2_, and lower systemic cardiac output. These interventions yielded immediate effect (SpO_2_ 86%–90%). In addition, we made efforts to increase oxygen‐carrying capacity and ensure adequate intravascular volume, through judicious transfusion of packed red blood cells (PRBCs). Although these efforts improved oxygenation, the patient ultimately succumbed to acute respiratory distress syndrome related to COVID‐19 with MSSA superinfection.

## 2 DISCUSSION

The strategies we used were intended to optimize oxygenation in a patient with severely limited pulmonary gas exchange. These include (A) capturing a greater fraction of the native cardiac output to route it through the VV‐ECMO circuit, (B) increasing SvO_2_ by lowering oxygen consumption, (C) increasing oxygen‐carrying capacity of the blood, and (D) improving pulmonary gas exchange.

### 2.1 How can the fraction of cardiac output routed through the VV‐ECMO circuit be determined and increased?

In our patient, oxygenation occurred entirely within the VV‐ECMO circuit. Thus, SaO_2_ depended on the fraction of systemic cardiac output reaching the VV‐ECMO circuit. When the VV‐ECMO blood flow is set and known, it is clinically important to estimate the systemic cardiac output in this setting to know the maximal improvement in oxygenation that can be provided by the circuit.

**FIGURE 3:**
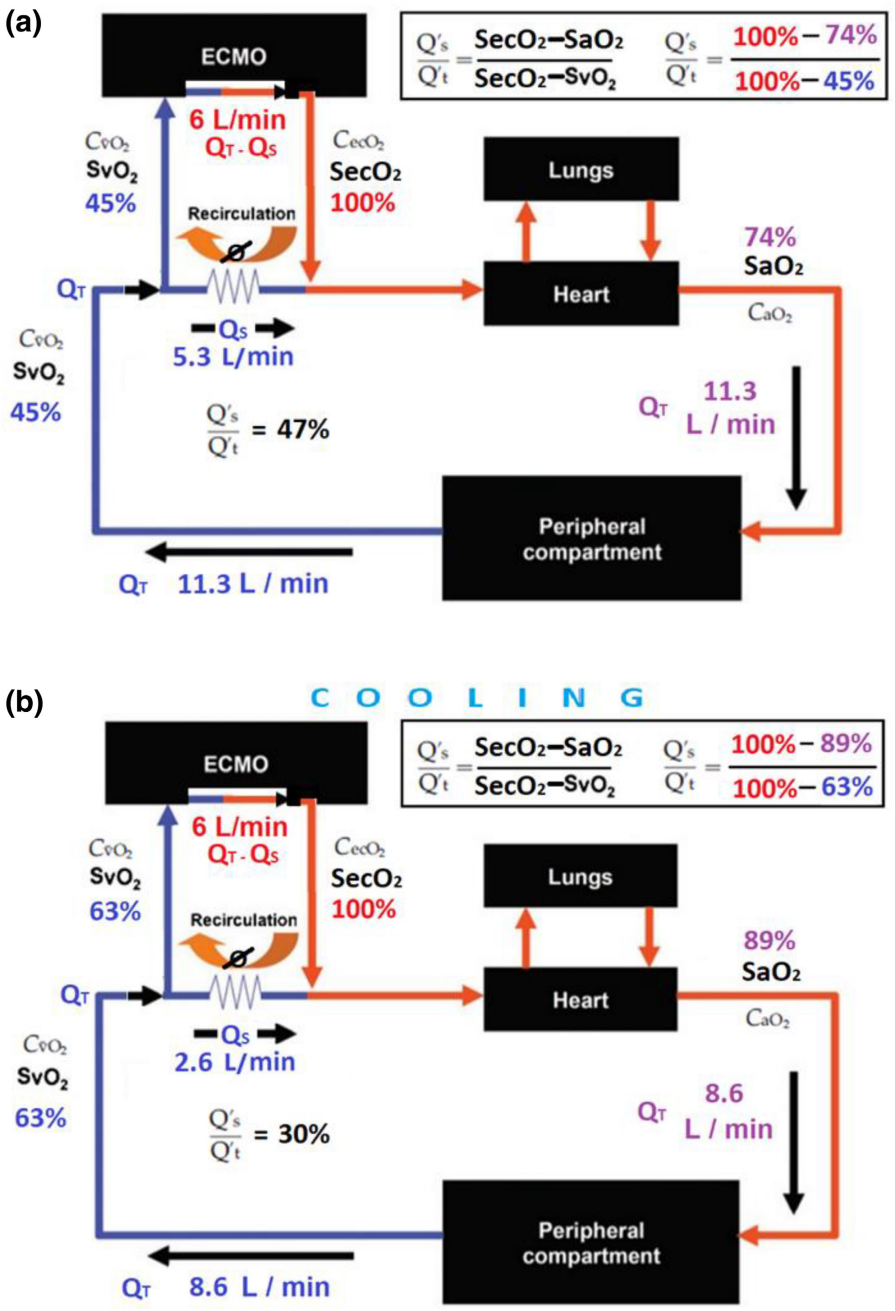
VV‐ECMO model. Panel (a) exemplifies the hyperdynamic, septic state with high cardiac output [Q_T_] of 11.3 L/min. In the setting of severe lung injury with complete pulmonary failure, oxygenation is dependent on ECMO blood flow (6 L/min). In this example, 5.3 L/min (47% of the venous return) will shunt [*Q*_S_] through the vena cava without oxygenation. With a shunt fraction [*Q*_S_/*Q*_T_] of 47% and a low SvO_2_ of 45%, the result is severe hypoxemia with an SaO_2_ of only 74%. Panel (b) exemplifies the effect of cooling on cardiac output [*Q*_T_] and SvO_2_. In the setting of severe lung injury with complete pulmonary failure, oxygenation is dependent on ECMO blood flow (6 L/min). After cooling by 1°C on unchanged ECMO settings (*compared to Panel a*) cardiac output [*Q*_T_] decreased to 8.6 L/min (from 11.3 L/min) and the shunt fraction [*Q*_S_/*Q*_T_] decreased to 30% (from 47%). SvO_2_ increased to 63% (from 45%) as a result from lower oxygen consumption from cooling.

When pulmonary gas exchange is negligible and oxygenation is provided entirely by the VV‐ECMO circuit, the a two compartment model of shunt physiology and the shunt equation can be used to calculate the systemic cardiac output [*Q*_T_] and shunt flow [*Q*_s_] (Figure 2; Petersson & Glenny, 2014). In VV‐ECMO, the native cardiac output [*Q*_T_] divides into two paths: (1) the systemic blood not drawn into the ECMO circuit that is unoxygenated shunt‐flow [*Q*_s_] and (2) ECMO‐circuit blood flow that is oxygenated [*Q*_T_ − *Q*_s_]. In this conceptual two‐compartment model, systemic oxygenation depends on the relationship between VV‐ECMO blood flow [*Q*_T_ − *Q*_S_] and systemic cardiac output [*Q*_T_] (Figure 2). With a known ECMO blood flow [*Q*_T_ − *Q*_S_], one can apply the original shunt equation to estimate the systemic cardiac output by making a couple of mathematical adjustments. (Figures 2 and 3; Table 2a): Beginning with the original shunt equation:

(1) $$Q_{s}/Q_{T}=\left({\text{CecO}}_{2}-{\mathrm{CaO}}_{2}\right)/\left({\text{CecO}}_{2}-{\mathrm{CvO}}_{2}\right)$$

where CecO_2_ is the oxygen content in maximally saturated post‐ECMO blood, CaO_2_ is the oxygen content of arterial blood and CvO_2_ is venous oxygen content. Ignoring dissolved oxygen, this can be simplified by calculating the shunt fraction based on oxygen saturations (Walley, 2011). As the saturation of post‐ECMO blood is 100%, the shunt equation simplifies to

(2) $$Q_{S}/Q_{T}=\left(100\%-{\mathrm{SaO}}_{2}\right)/\left(100\%-{\mathrm{SvO}}_{2}\right)$$

In our patient with a SaO_2_ of 74% and SvO_2_ of 45%, this equation predicts a shunt fraction of 47% (Table 2a and Figure 3a). Recall that the shunt fraction, in this case, represents the portion of the cardiac output that does not travel through the VV‐ECMO circuit. To determine the total cardiac output, we calculate

(3) $$\begin{array}{c} \text{Total Cardiac Output}\;Q_{T}=\\ \left(Q_{T}-Q_{S}\right)/\left(1-Q_{S}/Q_{T}\right);\left(\mathrm{see}\;\text{derivation in Table}\;2\right) \end{array}$$

**TABLE 2:** Clinical data and cardiac output calculations.

Parameter	(a) Day 11, 15:00	(b) Day 11, 21:00 cooling	(c) Day 13, 11:00	(d) Day 13, 11:00
Temperature (°C)	36.8	35.9	36.2	36.2
SaO_2_ (%)	74%	89%	81%	
SvO_2_ (%)	45%	63%	46%	
PecO_2_ (mmHg)	224	228	186	
Hgb (g/dL)	7.5	7.5	8.7	8.7
CaO_2_	7.55	9.097		
CvO_2_	4.62	6.43		
CecO_2_	10.72	10.74		
ECMO flow [=*Q*_T_ − *Q*_S_]	6 L	6 L	5.64 L	
*Q*_S_/*Q*_T_ = [(CecO_2_ − CaO_2_)/(CecO_2_ − CvO_2_)]	(10.72 − 7.55)/(10.72 − 4.62) = 0.52	(10.74 − 9.097)/(10.74 − 6.43) = 38		
*Q*_S_/*Q*_T_ = [(100% − SaO_2_)/(100% − SvO_2_)]	(1 − 0.74)/(1 − 0.45) = 0.47	(1 − 0.89)/(1 − 0.63) = 0.30	(1 − 0.81)/(1 − 0.46) = 0.35	
*Q*_T_ = [(*Q*_T_ − *Q*_S_)/(1 − *Q*_S_/*Q*_T_)]^a^	6 L/(1 − 0.47) = 11.3 L/min	6 L/(1 − 0.3) = 8.6 L/min	5.64 L/(1 − 0.35) = 8.7 L	
*Q*_s_ [=*Q*_T_ − (*Q*_T_ − *Q*_S_)]	5.3 L/min	2.6 L/min	3.045 L/min	
Aortic area				4.95 cm^2^
Velocity Time Integral (VTI)				20.69 cm/contraction
Stroke Volume [SV = VTI × area]				102 cm^3^
Heart Rate				89 bpm
Cardiac Output CO = SV × HR				102 cm^3^ × 89 = 9.07 L

Abbreviations: CaO_2_, oxygen content of arterial blood; CecO_2_, “endcapillary” content of oxygen = post‐ECMO‐oxygenator content of oxygen; CvO_2_, oxygen content of venous blood; PecO_2_, “endcapillary” partial pressure of oxygen = post‐ECMO‐oxygenator partial pressure of oxygen; SaO_2_, saturation of arterial blood with oxygen; SvO_2_, saturation of central venous blood with oxygen.

^a^ Derivation of equation to solve for *Q*_T_: *Q*_T_ = (*Q*_T_ − *Q*_S_) + *Q*_S_ => Dividing by *Q*_T_ => 1 = (*Q*_T_ − *Q*_S_)/*Q*_T_ + *Q*_S_/*Q*_T_ => Subtract *Q*_S_/*Q*_T_ from both sides => 1 − *Q*_S_/*Q*_T_ = (*Q*_T_ − *Q*_S_)/*Q*_T_ => multiply by *Q*_T_ => [1 − *Q*_S_/*Q*_T_] × *Q*_T_ = *Q*_T_ − *Q*_S_ => Dividing by [1 − *Q*_S_/*Q*_T_] = ≥*Q*_T_ = (*Q*_T_ − *Q*_s_)/(1 − *Q*_S_/*Q*_T_).

Based on a VV‐ECMO blood flow [*Q*_T_ − *Q*_S_] of 6 L/min and a shunt fraction [*Q*_S_/*Q*_T_] of 47%, we estimate the total cardiac output to be is 11.3 L/min (Figure 3a and Table 2a). This is consistent with hyperdynamic cardiac output seen in sepsis.

**FIGURE 4:**
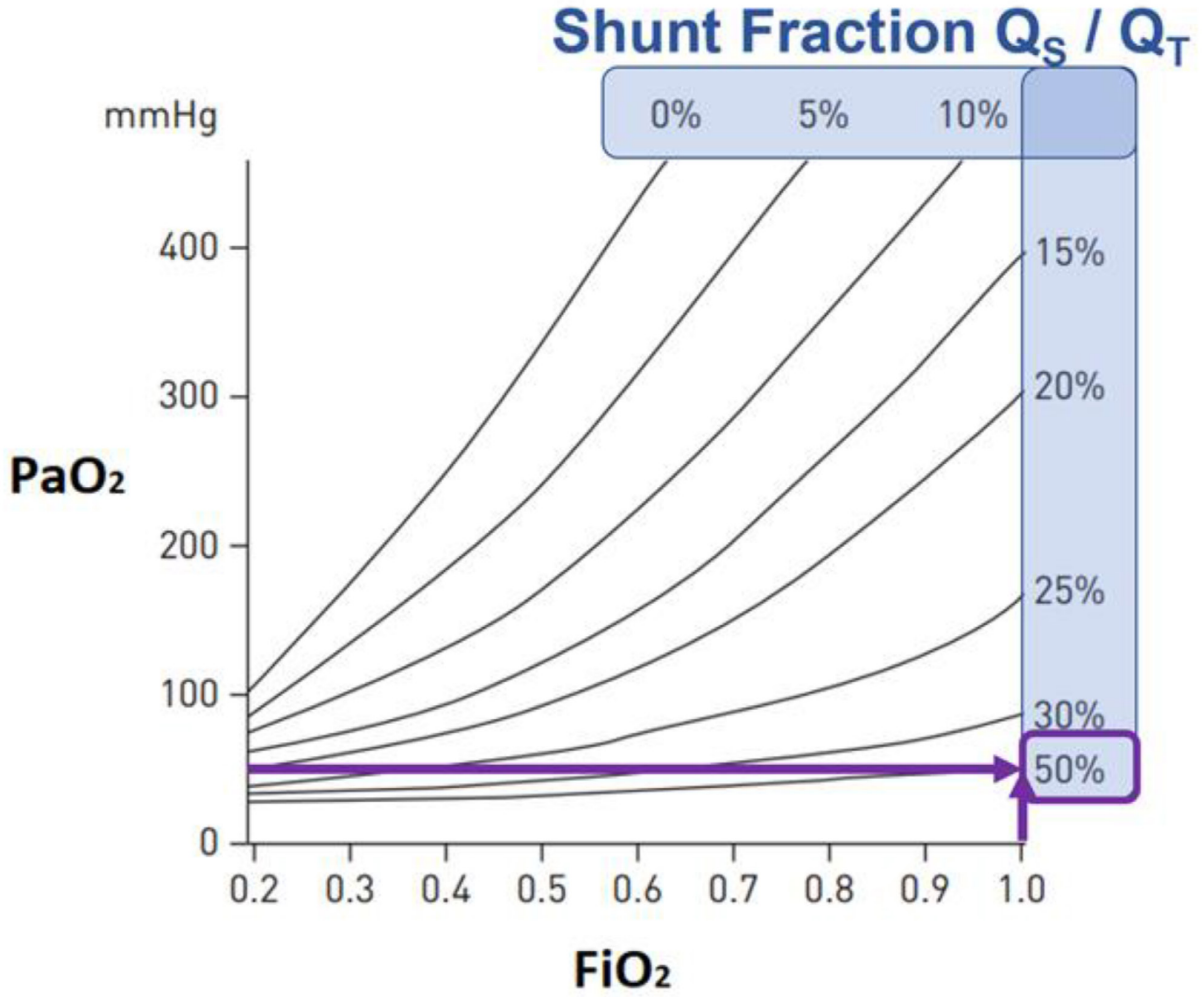
Iso‐shunt diagram, illustrating the relationship between PaO_2_ and FiO_2_ in the presence or absence of a shunt. In our patient, FiO_2_ is 100% via VV‐ECMO. The iso‐shunt lines correspond to different percentages of shunt flow in relation to systemic cardiac output (shunt fraction). In the absence of shunt, there is a near linear relationship between PaO_2_ and FiO_2_. With increasing shunt fractions, the change in PaO_2_ with increasing FiO_2_ is much flatter. If the shunt is ≥30% of cardiac output even a FiO_2_ of 1.0 results only in a PaO_2_ of <100 mmHg.

We can use this shunt estimate to predict the maximum ability to raise PaO_2_. Figure 4 shows an iso‐shunt diagram, which illustrates the relationship between PaO_2_ and FiO_2_ in the presence of a shunt (Petersson & Glenny, 2014). In our case of complete pulmonary failure on VV‐ECMO support, the FiO_2_ is the membrane oxygenator FiO_2_. The iso‐shunt lines correspond to different shunt fractions [*Q*_S_/*Q*_T_]. With increasing *Q*_S_/*Q*_T_, the effect of an increase in FiO_2_ is a progressively smaller increase in PaO_2_. When the *Q*_S_/*Q*_T_ reaches 50%, there is no significant increase in PaO_2_, even at a FiO_2_ of 1 (Figure 4). Thus, in our patient with a *Q*_s_/*Q*_T_ of 47% (Table 2a and Figure 3a) the best solution is to decrease *Q*_s_/*Q*_T_ by increasing the fraction of flow of systemic cardiac output through the VV‐ECMO circuit.

The shunt equation presents a valid approach to estimate systemic cardiac output based on the following observations: (A) There was no change in SaO_2_ or PaO_2_ in our patient when the ventilator FIO_2_ was titrated from 0.3 to 1 and endtidal CO_2_ was undetectable (Table 1). Our patient's complete pulmonary failure remained unchanged with ongoing “white out” on CXR (Figure 1) with a severely reduced respiratory system compliance of 3–4 mL/cm H_2_O. (B) There was no evidence of ECMO circuit recirculation and (C) a similar systemic cardiac output [*Q*_T_] measurement from transthoracic echo was confirmed on VV‐ECMO day#13.

From the transthoracic echo, the left ventricular outflow tract (LVOT) diameter was 2.51 cm and the velocity time integral (VTI) was 20.69 cm/contraction. Based on these measurements, the estimate of stroke volume (SV) was 102 mL:

$$\begin{array}{c} \mathrm{SV}={\text{Area}}_{\text{LVOT}}\times {\mathrm{VTI}}_{\text{LVOT}}\\ =4.95\;{\mathrm{cm}}^{2}\times 20.69\;\mathrm{cm}/\text{contraction}=102\;{\mathrm{cm}}^{3}\;\text{or}\;102\;\mathrm{mL} \end{array}$$

With a heart rate (HR) of 89 beats/min, the echo‐based estimate of systemic cardiac output was 9.07 L/min (Table 2d):

$$\begin{array}{c} \text{Cardiac Output}=\mathrm{SV}\times \mathrm{HR}=102\;\mathrm{ml}\times \;89=9.07\;L \end{array}$$

The systemic cardiac output [*Q*_T_] estimate based on VV‐ECMO (5.64 L/min ECMO blood flow, SvO_2_ 46%) and blood gas data (SaO_2_ 81%) at the time of the echo was 8.7 Lpm and the shunt fraction was 35% (Table 2c). This demonstrates that simple equation bedside estimates of shunt fractions are easy to calculate, and can be calculated frequently, from readily available information.

### 2.2 How was the fraction of the cardiac output through the VV‐ECMO circuit improved?

At maximal capacity, the VV‐ECMO circuit can accept and oxygenate 6 L/min of the cardiac output (Nunes et al., 2014). If the cardiac output is reduced, a larger fraction will flow through the ECMO circuit. Our sedated and paralyzed patient was cooled by approximately 1°C from a temperature range of 36.4–36.8°C to 35.3–35.9°C (Table 1). The shunt fraction decreased from 47% precooling to 30% postcooling, and estimated systemic cardiac output decreased from a 11.3 L/min precooling to 8.6 L/min postcooling (Tables 1 and 2b; Figure 3b). Because a greater fraction of the cardiac output flowed through the VV‐ECMO circuit, SaO_2_ increased from 73%–82% to 87%–89% and SvO_2_ increased from 44%–52% range to 56%–68% range.

### 2.3 How did cooling improve SvO_2_?

**FIGURE 5:**
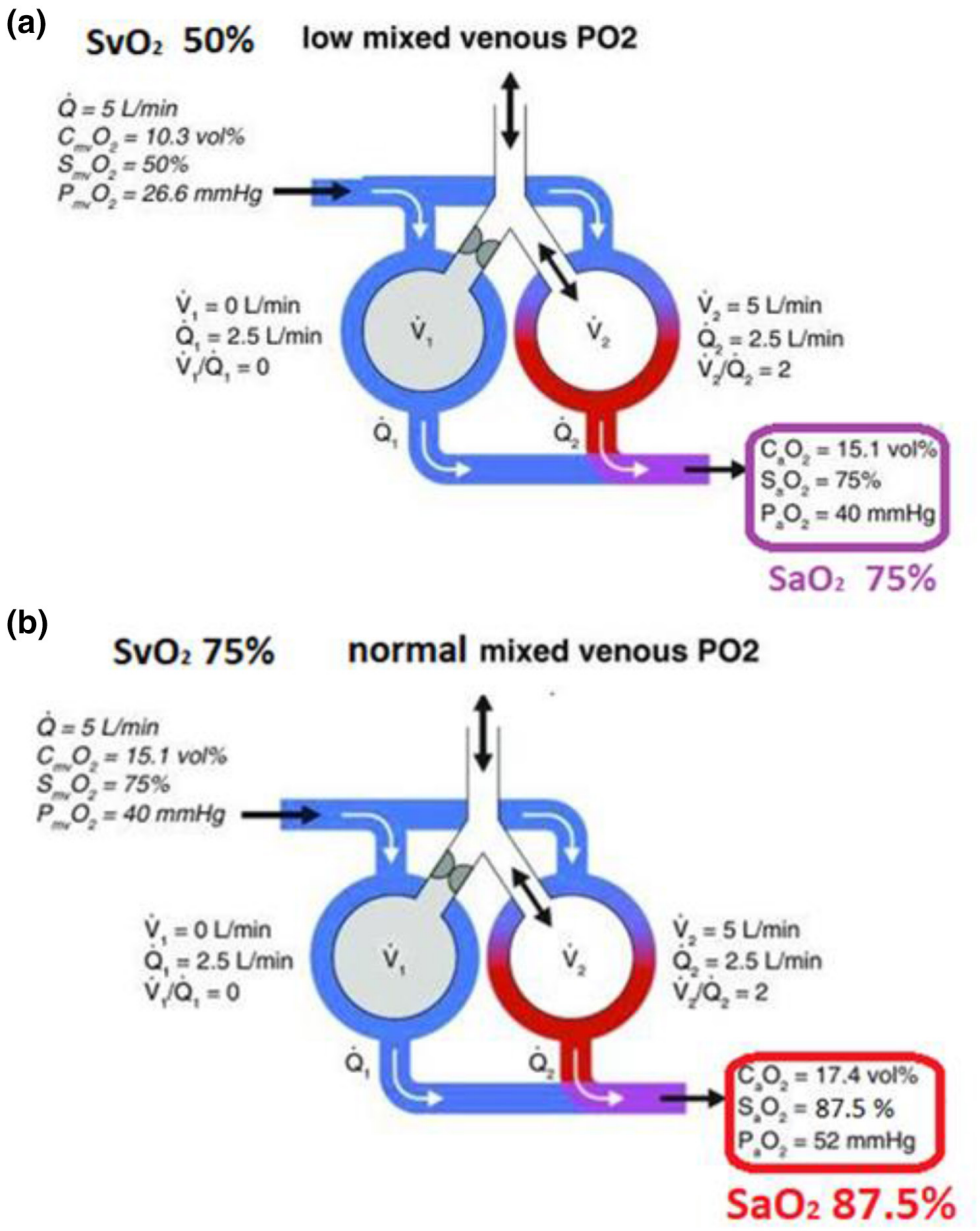
Impact of SvO_2_ on SaO_2_ in the setting of a significant shunt. In this example, the shunt fraction is 50%. Then, half of every increase in SvO_2_ will be reflected in SaO_2_. As shown in Figure 5, when SvO_2_ increases from (a) 50% to (b) 75% (Δ 25), then SaO_2_ increases from (a) 75% to (b) 87.5% (Δ 12.5) (Mishra et al., 2016; Walley, 2011).

In the setting of a significant shunt, SvO_2_ has a substantial impact on SaO_2_. This is illustrated in Figure 5. For example, when the shunt fraction is 50%, and then half of every increase in SvO_2_ will be reflected in an increase in SaO_2_. When SvO_2_ increases from 50% to 75% (Δ 25%), then SaO_2_ increases from 75% to 87.5% (Δ 12.5) (Mishra et al., 2016; Walley, 2011).

Another goal of cooling is to reduce oxygen consumption and subsequently increase SvO_2_ (Harris et al., 1971; Manthous et al., 1995). Previous work demonstrates that decreased body temperature is associated with decreased oxygen consumption (Harris et al., 1971; Manthous et al., 1995). At physiological temperatures, the relationships between metabolism, oxygen consumption, and hypothermia are particularly pronounced. Rather sharp decreases in oxygen consumption can occur even with small temperature changes. Our sedated and paralyzed patient was cooled by approximately 1°C, and his SvO_2_ increased from 45% to 63%, which is best explained by a decrease in oxygen consumption. The O_2_ extraction, as reflected in the SaO_2_‐SvO_2_ difference, was 29% precooling (36.8°C) and 26% postcooling (35.9°C). This 10% reduction in extraction is in line with the empirically derived 10% reduction in metabolism and O_2_ consumption per °C reduction in core temperature.

### 2.4 How was oxygen‐carrying capacity improved?

Our patient had a hemoglobin of 7.5 g/dL. Although there was no evidence of active bleeding and our patient did not meet the general threshold for a blood transfusion, we decided to transfuse 1 unit of packed red blood cells. We also raised the hemoglobin transfusion threshold to 8 g/dL. In addition to augmentation of oxygen delivery, the expansion of his intravascular volume via the PRBC administration maintained optimized ECMO blood flows.

Hemoglobin (Hgb) carries nearly all the oxygen in blood, except for a very small component of dissolved oxygen. The total oxygen content in the arterial blood is calculated by the equation:

$${\mathrm{CaO}}_{2}=\left[1.34\times \mathrm{Hgb}\times \left({\mathrm{SaO}}_{2}/100\right)\right]+\left[0.003\times {\mathrm{PaO}}_{2}\right]$$

Thus, modulating the concentration of hemoglobin has the greatest ability to alter blood oxygen content. In our patient, considering his marginal oxygen delivery (with an SaO_2_ as low as 75%), we decided to transfuse 1 unit of packed red blood cells and temporarily raised the hemoglobin goal to 8 g/dL. This improved oxygen delivery, even when saturation was suboptimal.

### 2.5 What attempts were made to improve pulmonary gas exchange?

The goal of ECMO is to allow a pathway to lung recovery by providing adequate oxygenation with ultra‐lung protective ventilation to avoid ventilator induced lung injury (Brodie et al., 2019). Furthermore, ECMO support can reduce the need for sedation and facilitate physical therapy. Despite ultra‐lung protective ventilation, recruitment maneuvers, and bronchoscopies for airway clearance, our patient continued to have complete pulmonary failure. Prone positioning on ECMO support was not possible, as even small positional changes compromised ECMO‐circuit flows with subsequent hypoxemia. Because of sepsis, medical instability, and obesity, ECMO could not provide a bridge to lung transplant. Despite improvements described in this presentation, our patient eventually died from irreversible pulmonary failure due to COVID pneumonia complicated by staphylococcus aureus sepsis (Kurihara et al., 2022).

## 3 SUMMARY

In patients with complete pulmonary failure on VV‐ECMO, the “shunt analogy” illustrates the relationship between the two circulations: ECMO circuit blood flow and patient cardiac output. Oxygen delivery depends on the relationship between VV‐ECMO blood flow [*Q*_S_ − *Q*_T_] to systemic cardiac output [*Q*_T_]. The physiological principal to estimate systemic cardiac output [*Q*_T_] is based on the shunt equation. With this simple equation in mind, bedside estimates of shunt fractions are easy to calculate from readily available information. Interventions to lower the shunt fraction [*Q*_S_/*Q*_T_] and to increase SvO_2_, like cooling, increase SaO_2_ and oxygen delivery.

## AUTHOR CONTRIBUTIONS

RS and ME involved in concept and drafting of manuscript. AS, RMR, JH, MB, and GS involved in revision and editing of manuscript.

## FUNDING INFORMATION

No funding information provided.

## ETHICS STATEMENT

The manuscript is a retrospective case report that does not require ethics committee approval at that institution.
